# Dataset on the effects of environmentally relevant humic acid concentrations on the liver protein profile in Japanese medaka (*Oryzias latipes*)

**DOI:** 10.1016/j.dib.2022.107796

**Published:** 2022-01-05

**Authors:** Victoria V. Yurchenko, Alexey A. Morozov, Roman A. Fedorov, Ludmila G. Bakina, Victor G. Zgoda, Olga V. Tikhonova

**Affiliations:** a Papanin Institute for Biology of Inland Waters Russian Academy of Sciences, 109, IBIW RAS, Borok 152742 Russia; b St Petersburg Federal Research Center of the Russian Academy of Sciences, 14th Line, 39, SPC RAS, Saint Petersburg 199178 Russia; c Institute of Biomedical Chemistry, Pogodinskaya St., 10, IBMC, Moscow 119121 Russia

**Keywords:** Humic substances, Acute exposure, Japanese rice fish, Shotgun proteomics, LFQ

## Abstract

The data were obtained by a label-free quantification approach from a shotgun proteomics experiment, using STrap sample processing technique for protein digestion and high-performance liquid chromatography with tandem mass spectrometry (HPLC-MS/MS) for peptide analysis. MaxQuant data processing was used to obtain proteomics data. The dataset reflects changes in the liver protein profile of Japanese medaka exposed to 0, 5, 40 and 80 mg/L nominal concentrations of Sigma-Aldrich humic acid for 96 h. Actual concentrations of humic acid were measured using the potassium dichromate photometric method and reported in mg organic carbon/L. These proteomics data are relevant for further insights into fish stress responses to humic substances-related challenge.

## Specifications Table

**Table:** 

Subject	Omics: Proteomics
Specific subject area	Acute stress response in fish
Type of data	Spreadsheets Table
How data were acquired	S-Trap sample processing (ProtiFi); HPLC-MS/MS analysis (Thermo Scientific UltiMate 3000 RSLCnano system; Thermo Scientific Q Exactive HF-X hybrid quadrupole-Orbitrap mass spectrometer); protein identification with MaxQuant software (version 1.6.3.4) and protein sequences of the *Oryzias latipes* proteome provided by UniProt (Feb 2021).
Data format	Raw Filtered
Parameters for data collection	Fish were exposed to 0, 5, 40 and 80 mg/L humic acid for 96 h. Pooled liver samples from 8 fish, four males and four females, per treatment group were prepared. Three technical replicates of each sample were analysed using HPLC-MS/MS.
Description of data collection	Medaka was exposed to humic acid. Fish livers were homogenised, homogenate aliquots were pooled. Then, pooled samples were frozen and stored in liquid nitrogen. Protein digestion was performed using S-Trap sample processing technology. The resulting peptides were analysed by HPLC-MS/MS method. Mass spectrometry data were processed with the MaxQuant software with the built-in Andromeda peptide search engine, using the *Oryzias latipes* protein sequences provided by the UniProt Knowledgebase.
Data source location	Institute of Biomedical Chemistry, Moscow, Russia
Data accessibility	Raw mass-spectrometry data were deposited to the ProteomeXchange Consortium via the jPOST partner repository [1]; the accession numbers are PXD025749 for ProteomeXchange and JPST001150 for jPOST (https://repository.jpostdb.org/entry/JPST001150). MaxQuant output is available with the article (Supplementary data).
Related research article	V. Yurchenko, A. Morozov, Responses of hepatic biotransformation and antioxidant enzymes in Japanese medaka (*Oryzias latipes*) exposed to humic acid, Fish Physiol. Biochem. https://doi.org/10.1007/s10695-021-01034-4[2]

## Value of the Data

• These data show changes in the fish liver proteome in response to a short-term humic substances-related challenge.
• Researchers in the fields of fish physiology and aquaculture can benefit from these data.
• The data might be reused for further research of fish stress and welfare in a humic environment.

## 1 Data Description

The dataset is comprised of proteomics data from four pooled liver samples obtained in the laboratory experiment described below. Measured organic carbon concentrations (mg/L) and pH values of tested solutions are given in Table 1. The dataset presents data obtained by the HPLC-MS/MS analysis. Mass spectrometry data were deposited to the ProteomeXchange Consortium via the jPOST partner repository. The output from a MaxQuant software is given as a Microsoft Excel workbook, as raw and filtered data (Supplementary data, proteinGroupsLiver_ORYLA.xlsx). The “proteinGroups” sheet contains the raw MaxQuant output: all the distinct proteins identified and quantified. The “proteinGroups_filtered” sheet presents only the proteins reliably identified by minimally two peptides. The instrument settings for the proteomics analysis are presented as separate files (Supplementary data, parameters.txt and summary.txt). The “LFQ_CV values” sheet contains calculations of the coefficient of variation of the label-free quantitation (LFQ) values for each identified protein group between the technical replicates. In the “LFQ_Mean CV” sheet, mean values of the coefficient of variation are calculated to estimate the variation between technical replicates.

**Table 1:** Nominal humic acid and measured organic carbon concentrations (mg/L) and pH values of tested solutions.

Humic acid	OC a, 0 h	OC, 78 h	pH b
0	<LOQ c	<LOQ	8.14 ± 0.16
5	<LOQ	<LOQ	7.98 ± 0.43
40	4.5	6.6	8.10 ± 0.24
80	9.4	9.4	8.16 ± 0.22

a OC – organic carbon; concentrations are given as means (*n* = 2 per sample)

b pH values throughout the exposure period, given as means ± SD

c LOQ – limit of quantitation

## 2 Experimental Design, Materials and Methods

### 2.1 Fish, exposure and sampling

The breeding stock of Japanese medaka, *Oryzias latipes* (Temminck & Schlegel, 1846), Hd-rR strain, has been maintained in the Laboratory of Physiology and Toxicology of Aquatic Animals (IBIW RAS) for five years. A stock intended for the humic acid exposure was held for one month under semi-static conditions similar to those set during the exposure: 50% daily water renewal, water temperature 25±1°C and 16:8 h photoperiod. Aerated tap water from the groundwater source was used (conductivity 462±6 µS/cm, pH 7.97-8.12), and the loading was kept at ≤ 1 g wet weight of fish per litre.

Adult medaka (42-44 weeks old, 0.48 ± 0.06 g wet weight) were exposed to 0, 5, 40, and 80 mg/L nominal concentrations of humic acid (CAS Number 1415-93-6, Sigma-Aldrich) for 96 h. The testing procedure followed the OECD Test Guideline No. 203: Fish, Acute Toxicity Testing [3], except for the feeding regime (given below). During the exposure period, pH values in test chambers were measured daily in duplicate (Table 1).

On day 0 of the experiment, testing solutions were prepared by dissolving the needed amount of dry humic acid in 10 L of water. For example, 0.8 g of humic acid was triturated in a bowl, dissolved in a small volume of water and transferred into a graded tank. The final volume was brought up to10 L to obtain an 80 mg/L testing solution. Testing solutions for renewal were prepared daily by serial dilution of an 80 mg/L solution, as described above. According to the product specification sheet, the Sigma-Aldrich humic acid is soluble in water (0.1 g in 10 mL) and turbid (i.e. contains insoluble matter in a solution).

For the exposure, test chambers were filled with 10 L of the solutions, and the loading of fish was ≤ 0.5 g/L. Although the main idea is that the fish should be randomly distributed among treatments, a count of males and females placed in each chamber was kept to achieve an equal proportion.

Fish were fed commercial food (TetraMin Mini Granules) four times per day using automatic feeders and *Artemia nauplii* twice a day manually. Residual food granules and faeces were removed daily during the water renewal.

At the end of the exposure period, after anesthetisation with tricaine methanesulfonate (MS-222, 100 mg/L), the fish was weighed and dissected, and the liver was excised on ice, weighed and placed into a 200 µL centrifuge tube. Ice cold phosphate-buffered saline was added in a 1:3 ratio (weight/volume), and a sample was then homogenised on ice for 3 min with a manual pestle. Homogenate aliquots from 8 individual liver samples (from 4 males and 4 females) in each treatment group were pooled and frozen in liquid nitrogen to be used later for the proteomics analysis.

### 2.2 Organic carbon analysis

In semi-static renewal experiments, test concentrations should be measured at least twice over one exposure period (before and after the renewal of test solutions) [3]. Therefore, test solutions were measured at 0 h (before distributing fish among treatments) and 78 h (6 h after the third renewal of test solutions). Humic acid solutions were analysed in duplicate. Water samples were centrifuged at 10,000 g for 5 min to separate the non-dissolved and any other interfering particles, and supernatants were used for the analysis. Organic carbon concentrations were measured employing the potassium dichromate photometric method using a KFK-3 instrument (ZOMZ, Russia) [4].

### 2.3 Label-free protein quantification and identification of proteins

Each sample pre-treated with 5% sodium dodecyl sulfate (SDS) solution was ultrasonicated for 1 min at 4°C. Then, total protein concentration was measured employing the bicinchoninic acid assay using 0.5, 1, 2, 5, 10, 25, and 50 µg/µL bovine serum albumin (BSA) solutions to plot a standard curve. To 30 µL of sample or BSA solution, a 1 mL working solution was added (1% water solution of bicinchoninic acid disodium salt, 2% sodium carbonate, 0.16% sodium tartrate, 0.4% sodium hydroxide, 0.95% sodium bicarbonate, and 4% copper(II) sulfate, pH 11.25). The reaction mixture was then incubated at 56°С for 20 min using a Termomixer comfort shaker (Eppendorf, Germany). Measurements were carried out in triplicate using a NanoDrop ND-1000 spectrophotometer (Thermo Fisher Scientific, USA) at λ = 562 nm.

Trypsin protein digestion was performed by the suspension trapping (STrap) sample preparation method [5] using S-Trap Mini Spin Columns (ProtiFi, USA) according to the manufacturer's manual. To reduce and alkylate disulfide bonds, the samples were incubated with 4mM tris(2-carboxyethyl)phosphine and 6.2 mM 2-chloroacetamide at 80°С for 30 min. Then the samples were cooled to room temperature. To denature proteins completely, 12% orthophosphoric acid was added. After that, the S-Trap protein binding/washing buffer (100 mM triethylammonium bicarbonate in 90% methanol, pH 7.55) was added to the samples. Samples were mixed and transferred to the S-Traps. Then, S-Trap columns were centrifuged at 4,000 *g* for 4 min to trap proteins (repeated if necessary, until all solutions had passed through). Protein cleaning was performed by repeatedly adding the same S-Trap protein binding/washing buffer and centrifuging at 4000 g for 4 min to remove the buffer entirely. S-trap columns were then transferred to clean sample tubes for digestion, and the digestion buffer containing protease (50 mM triethylammonium bicarbonate, trypsin) was added to the top of each S-Trap. Samples were incubated at 47°С for 90 min. After that, to elute peptides for analysis, the elution buffer 1 (50 mM triethylammonium bicarbonate in water), buffer 2 (0.2% formic acid in water) and buffer 3 (50% acetonitrile in water) were added, and samples were centrifuged at 4000 g for 4 min after adding each buffer. Eluted peptides were pooled, dried down and resuspended in 0.1% formic acid.

Further analysis of obtained peptides was performed by high-performance liquid chromatography with tandem mass spectrometry (HPLC-MS/MS) described below. A 1 µg aliquot of peptides in a volume of 1-4 µL was loaded onto the Acclaim µ-Precolumn (0.5 × 3 mm, 5 µm particle size, Thermo Scientific) at a flow rate of 10 µL/min for 4 min in an isocratic mode of Mobile Phase C (2% acetonitrile, 0.1% formic acid). Then, the peptides were separated with HPLC (Thermo Scientific UltiMate 3000 RSLCnano system, Rockwell, IL, USA) in a 15-cm long C18 column (Acclaim Pep-Map RSLC inner diameter of 75 µm, Thermo Fisher Scientific, Rockwell, IL, USA). The peptides were eluted with a gradient of buffer B (80% acetonitrile, 0.1% formic acid) at a flow rate of 0.3 µL/min. Total run time was 90 min, which included initial 4 min column equilibration to buffer A (0.1% formic acid), a gradient from 5 to 35% of buffer B for 65 min, then 6 min to reach 99% of buffer B, 10 min flushing with 99% of buffer B, and 5 min re-equilibration to buffer A. The MS analysis was performed in triplicate using a Thermo Scientific Q Exactive HF-X hybrid quadrupole-Orbitrap mass spectrometer (Rockwell, IL, USA). The capillary temperature was 240°C, and the voltage at the emitter was 2.1 kV. Mass spectra were acquired at a 120,000 resolution in a range of 300−1500 m/z. Tandem mass spectra of fragments were acquired at a 15,000 resolution in a range from 100 m/z to the value determined by a charge state of the precursor (not higher than 2000 m/z). The maximum integration time was 50 ms/110 ms for precursor and fragment ions, correspondingly. AGC (automatic gain control) target values for precursor and fragment ions were 1E6 and 2E5, correspondingly. An isolation intensity threshold of 50,000 counts was determined for precursor selection, and up to the top 20 precursors were chosen for fragmentation with HCD (higher-energy collisional dissociation) at normalised collision energy (NCE) of 29. Precursors with a charge state of 1+ and more than 5+ were rejected, and all measured precursors were dynamically excluded from triggering a subsequent MS/MS for 20 s.

Mass spectrometry data were processed using the MaxQuant software (version 1.6.3.4) with the built-in Andromeda peptide search engine. Protein sequences for the *Oryzias latipes* proteome provided by UniProt (Feb 2021) were used for protein identification. Carbamidomethylation of cysteines was set as fixed modification, and oxidation of methionines was set as a variable modification for the peptide search. A maximum m/z deviation of 4.5 ppm was allowed for precursor identification, and 20 ppm were set as match tolerance for fragment identification (acquisition in Orbitrap). One missed cleavage was allowed for trypsin digestion. The software option “Match between runs” was enabled, and features within a time window of 2 min were used to match between runs. The false discovery rates for peptide and protein identifications were set to 5%. Only proteins with minimally two peptides detected were considered reliably identified. Both sections of the UniProt Knowledgebase were used for protein identification: Swiss-Prot (manually annotated and reviewed) and TrEMBL (automatically annotated).

## Ethics Statement

Data collection complied with the ARRIVE guidelines and was carried out in accordance with the U.K. Animals (Scientific Procedures) Act, 1986 and associated guidelines, EU Directive 2010/63/EU, for animal experiments.

## CRediT authorship contribution statement

**Victoria V. Yurchenko:** Conceptualization, Investigation, Project administration, Writing – original draft, Writing – review & editing. **Alexey A. Morozov:** Investigation, Writing – original draft. **Roman A. Fedorov:** Investigation. **Ludmila G. Bakina:** Investigation. **Victor G. Zgoda:** Investigation, Writing – original draft, Formal analysis. **Olga V. Tikhonova:** Data curation, Writing – original draft.

## Declaration of Competing Interest

The authors declare that they have no known competing financial interests or personal relationships which have or could be perceived to have influenced the work reported in this article. The funders had no role in the experimental design, collection and analysis of the data.

## Appendix Supplementary materials

Image, application 1

Image, application 2

Image, application 3
